# Long-Term Effect of Manure and Fertilizer on Soil Organic Carbon Pools in Dryland Farming in Northwest China

**DOI:** 10.1371/journal.pone.0056536

**Published:** 2013-02-20

**Authors:** Enke Liu, Changrong Yan, Xurong Mei, Yanqing Zhang, Tinglu Fan

**Affiliations:** 1 Institute of Environment and Sustainable Development in Agriculture, Chinese Academy of Agricultural Sciences, Beijing, China; 2 Key Laboratory of Dryland Farming g Agriculture, Ministry of Agriculture of the People’s Republic of China (MOA), Beijing, China; 3 Dryland Agricultural Institute, Gansu Academy of Agricultural Sciences, Lanzhou, Gansu, China; Dowling College, United States of America

## Abstract

An understanding of the dynamics of soil organic carbon (SOC) as affected by farming practices is imperative for maintaining soil productivity and mitigating global warming. The objectives of this study were to investigate the effects of long-term fertilization on SOC and SOC fractions for the whole soil profile (0–100 cm) in northwest China. The study was initiated in 1979 in Gansu, China and included six treatments: unfertilized control (CK), nitrogen fertilizer (N), nitrogen and phosphorus (P) fertilizers (NP), straw plus N and P fertilizers (NP+S), farmyard manure (FYM), and farmyard manure plus N and P fertilizers (NP+FYM). Results showed that SOC concentration in the 0–20 cm soil layer increased with time except in the CK and N treatments. Long-term fertilization significantly influenced SOC concentrations and storage to 60 cm depth. Below 60 cm, SOC concentrations and storages were statistically not significant between all treatments. The concentration of SOC at different depths in 0–60 cm soil profile was higher under NP+FYM follow by under NP+S, compared to under CK. The SOC storage in 0–60 cm in NP+FYM, NP+S, FYM and NP treatments were increased by 41.3%, 32.9%, 28.1% and 17.9%, respectively, as compared to the CK treatment. Organic manure plus inorganic fertilizer application also increased labile soil organic carbon pools in 0–60 cm depth. The average concentration of particulate organic carbon (POC), dissolved organic carbon (DOC) and microbial biomass carbon (MBC) in organic manure plus inorganic fertilizer treatments (NP+S and NP+FYM) in 0–60 cm depth were increased by 64.9–91.9%, 42.5–56.9%, and 74.7–99.4%, respectively, over the CK treatment. The POC, MBC and DOC concentrations increased linearly with increasing SOC content. These results indicate that long-term additions of organic manure have the most beneficial effects in building carbon pools among the investigated types of fertilization.

## Introduction

Soil organic matter (SOM) plays a key role in the improvement of soil physical, chemical and biological properties [1]. Conservation of the quantity and quality of soil organic matter (SOM) is considered a central component of sustainable soil management and maintenance of soil quality [2]. Organic manure and inorganic fertilizer are the most common materials applied in agricultural management to improve soil quality and crop productivity [3]. Many studies have shown that balanced application of inorganic fertilizers or organic manure plus inorganic fertilizers can increase SOC and maintain soil productivity [4]–[7].

However, SOM is not sensitive to short-term changes of soil quality with different soil or crop management practices due to high background levels and natural soil variability [8]. Labile soil organic carbon pools like dissolved organic C (DOC), microbial biomass C (MBC), and particulate organic matter C (POC) are the fine indicators of soil quality which influence soil function in specific ways (e.g., immobilization–mineralization) and are much more sensitive to change in soil management practices [9], [10]. Because these components can respond rapidly to changes in C supply, they have been suggested as early indicators of the effects of land use on SOM quality [11]. Recently, many studies have reported responses of labile SOC pools to management practices [5], [12], [13], though limited to tillage practices or cropping intensity and rotations management [14]. Few studies have focused on the effect of labile organic C after long-term fertilizer application in northwest China.

In most cases, studies for SOC and SOC fractions have mostly focused on shallow surface soil [15]. The limited information on soil profile SOC and its fractions distribution is a hindrance to conclusive identification of beneficial effects after long-term fertilizer application. Thus, further research is needed to clarify fertilizer application impacts on SOC and SOC fractions for the entire soil profile. Documenting increased SOC levels at deeper depths in the soil profile, however, has been difficult due to a lack of studies where sampling occurred below 30 cm. Nayak et al. [13] found that applications of combined inorganic fertilizers with or without manure can sequester carbon in the 0–60 cm soil layer at the Indian sub-Himalayas. In hot humid subtropical eastern India, Majumder et al. [16] found that after 19 y in a puddle rice-wheat (Triticum aestivum L.) system, NPK+FYM treated plots had 14% larger labile C pools compared with the control plots in the 0–60 cm soil layer.

Northwest China is a vast semi-arid area with average annual precipitation ranging from 300 to 600 mm and more than 90% of the cropland depends on rain fall. Dryland farming has prevailed for several decades in this region. The dry climate and sparse vegetation are mainly responsible for the low SOM. A long period of cultivation and severe erosion in northwest China are likely other potential causes of low SOM [17]. SOC content in the 0–20 cm soil layer of this region is about 11.4 t C ha^–1^ [18]. In recent years, there has been a large increase in the use of inorganic fertilizer with a concomitant decrease in the use of manure. Challenges for dryland farming in Northwest China are low SOC and nutrient retention [19]. However, little is known about the long-term application of inorganic fertilizers either alone or with organic manure on SOC and the distribution of labile organic C fractions at different profile depths. Thus, it is crucial to collect SOC data from long-term experiments in order to understand and estimate the contribution of manure and fertilizer to soil C dynamics. This study provided a unique opportunity to examine the long-term effects of manure and fertilizer on soil organic carbon pools for dryland farming in Northwest China. We hypothesized that long-term fertilizer and manure application would influence the SOC and labile carbon. Moreover, we considered labile organic C fractions would be responsive indicators to SOC change with long histories of fertilizer managements. Our objective was to study the changes of the depth distribution (0–100 cm) in SOC and SOC fractions under a 30-year field experiment in the north of China, and to explain the relationship between different SOC fractions and SOC concentrations. Improved understanding of labile organic matter fractions will provide valuable information for establishing sustainable fertilizer management systems to maintain and enhance soil quality.

## Materials and Methods

### Experimental Site

**Figure 1:**
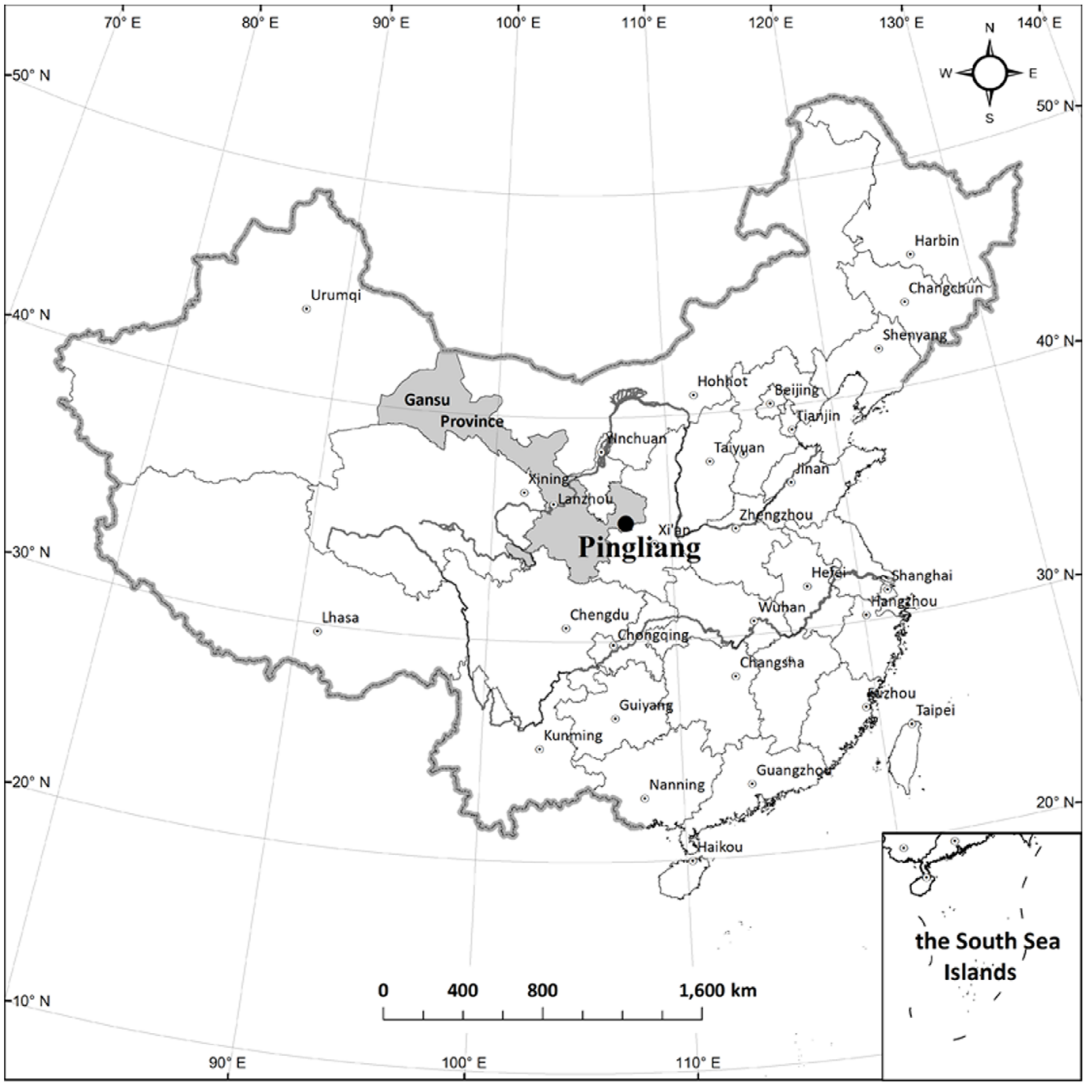
Location of the study site.

The research was based on a long-term fertilizer experiment started in 1979 at the Gaoping Agronomy Farm (35°16′N, 107°30′E, 1254 m altitude), Pingliang, Gansu, China (Fig. 1). Under average climatic conditions, the area has an aridity index (P/PET: precipitation/potential evapotranspiration) of 0.39 and receives 540 mm precipitation, about 60% of which occurs in the summer from July through September. May through June is the driest period for crop growth and little precipitation occurs during the winter months of December and January. The mean annual temperature is 9.8°C. The mean annual sunshine period is 2834 h. The soil is a dark loessial soil classified as calcarid regosols [20]. Analysis of soil samples taken from the experimental area in October 1978 indicated that the surface 15 cm of soil had a pH of 8.2, SOC content of 6.2 g kg^−1^, total N of 0.95 g kg^−1^, total P content of 0.57 g kg^−1^, available P of 7.2 mg kg^−1^ and available K of 165 mg kg^−1^.

### Experimental Design and Treatments

The experiment began in 1979 with a maize crop on land that had been cropped to maize during the previous year, one crop per year. Six fertilization treatments were arranged in a randomized complete block design with three replications. Maize was grown in 1979 and 1980, wheat from 1981 to 1984, maize in 1985 and 1986, wheat from 1987 to 1990, maize in 1991 and 1992, wheat from 1993 to 1998, soybean (Glycine max (L.) Merr.) in 1999, sorghum (Sorghum bicolor (L.) Moench) in 2000, wheat from 2001 to 2004, maize in 2005 and 2006, and wheat in 2006 to 2008.

Winter wheat (Qingxuan 8271, Longyuan 935, and Ping 93-2) was seeded in rows 14.7 cm apart at rates of 165 kg ha^−1^ on about 20 September each year when wheat followed wheat, and in early October when wheat followed maize. Maize was seeded about 20 April each year that maize was grown and Zhongdan 2 was seeded by hand in clumps every 33 cm in rows 66.5 cm apart. About 3 weeks after seeding, maize plants were thinned to one plant per clump. Later, if tillers developed, they were removed to avoid competition. Hand weeding was done to control weeds and plant protection measures were applied when needed. Crops were harvested manually close to the ground and all harvested biomass was removed from the plots. Grain yields were determined by harvesting 20 m^2^ for wheat and 40 m^2^ for maize at centers of the plots. Grain samples were air-dried on concrete, threshed, and oven-dried at 70°C for 48 hrs, and then weighed.

The experimental area was 0.44 ha. Each plot was 16.7 m×13.3 m with a buffer zone of 1.0 m between each plot. The six treatments were (1) CK, unfertilized control, (2) N, nitrogen fertilizer annually, (3) NP, nitrogen and phosphorus (P) fertilizers annually, (4) NP+S, straw (S) plus N added annually and P fertilizer added every second year, (5) FYM, farmyard manure added annually, and (6) NP+FYM, farmyard manure plus N and P fertilizers added annually. Urea was the N source and was applied to supply 90 kg N ha^−1^ yr^−1^. Superphosphate was the P source and applied to supply 30 kg P ha^−1^ yr^−1^. Farmyard manure was added at rate of 75 t ha^−1^ (wet weight). Deep plowing of approximately 23 cm was performed in July after wheat harvest or in October after maize harvest except for the years in which wheat followed maize. In those years, shallow disk tillage was done after maize harvest and wheat was seeded immediately.

Generally, the farmyard manure was a mixture of about 1∶5 ratio of wet cattle manure to loess soils and so its nutrient content was quite variable from year to year. The SOC, N, P, and K contents of manure were 11.37, 1.07, 0.69, and 12.3 g kg^−1^ in dry weight, indicating that manure is very low in N, and high in P and K. Although the specific amounts of nutrients added with manure each year were not determined, an application of approximately 75 t ha^−1^ (wet weight) supplied roughly 425 kg C ha^−1^, 40 kg N ha^−1^, 26 kg P ha^−1^, and 460 kg K ha^−1^ in manure annually to crops. For NP+S treatment, mean 5.61 t ha^−1^ y^−1^ of straw (winter wheat and maize) approximately 10 cm in length was returned to the soil prior to plowing, and P fertilizer was added every second year. The straw contained 2.45 t C ha^−1^, 29.8 kg N ha^−1^, 7.4 kg P ha^−1^, and 34.0 kg K ha^−1^.

### Soil Sampling

For SOC trend (0–20 cm layer )study from 1979 to 2008, the composite soil sample (0–20 cm) for each plot was prepared by mixing ten soil cores (4-cm inner diameter) collected randomly after the harvest during 1979 through 1991 and 1996 through 2008 at about 15 d after harvest. The fresh soil was mixed thoroughly, air dried for 7 d, sieved through a 2.0 mm sieve at field moisture content, mixed, and stored in sealed plastic jars for analysis. Sub-samples were drawn to determine SOC in the 0–20 cm soil layer. Soil organic C was not determined during 1992 through 1995.

For the distribution of SOC and labile organic C fractions at different profile depths study, soil profile samples (0–20, 20–40, 40–60, 60–80, and 80–100 cm) were collected from six treatments before wheat sowing in September 2008. In each plot the soil was collected from ten points randomly, and mixed into one sample. After carefully removing the surface organic materials and fine roots, each mixed soil sample was divided into two parts. One part of the soil sample was air-dried for the estimation of soil chemical properties and the other part was sieved through a 2 mm wide screen and immediately transferred to the laboratory for biochemical analysis. Soil fresh samples were kept at 4°C in plastic bags for a few days to stabilize the microbiological activity and analyzed within 2 weeks.

### Soil Analyses

Soil organic C and bulk densities measured using the method of Blake [21]. DOC was measured using the method of Jiang et al. [22]. POM-C was determined by the method of Cambardella and Elliott [23]. MBC was estimated by fumigation-extraction [24].

### Carbon Inputs

To compute the fraction of added C stabilized cumulative C input was estimated from exogenous supply of C to the soil through straw and FYM and plant input of C through root biomass, stubble and rhizodeposition (Table 1).

**Table 1:** Estimated mean annual crop biomass and carbon input (Mg C ha^−1^ yr^−1^) to soil under different fertilizer treatments.

Treatments	Mean annual crop biomass (Mg C ha^−1^ yr^−1^)					Mean annual carbon input (Mg C ha^−1^ yr^−1^)					
	grainyield	straw biomass	root biomass	stubble biomass	Rhizodeposition	Straw-C	Roots-C	Stubble-C	Rhizodeposition-C	FYM-C	Total C
CK	2.11	2.92	0.71	0.29	0.73	0.00	0.29	0.13	0.27	0.00	0.69
N	2.76	3.71	1.09	0.37	0.84	0.00	0.44	0.16	0.35	0.00	0.95
NP	4.59	5.58	1.46	0.56	1.22	0.00	0.59	0.24	0.50	0.00	1.34
FYM	4.33	5.15	1.48	0.52	1.19	0.00	0.60	0.22	0.47	0.43	1.72
NP+S	4.88	5.61	1.68	0.56	1.27	2.45	0.68	0.24	0.52	0.00	3.89
NP+FYM	5.49	6.63	1.96	0.66	1.45	0.00	0.80	0.29	0.60	0.43	2.11

The straw and stubble were collected from three 6 m^2^ areas for each plot immediately after the harvest of the grains in 1997 and 2005. The straw, stubble and samples were then oven-dried at 60°C for 72 h and weighed. The straw contributed 56.1, 55.6, 54.0, 54.2, 53.0 and 54.6 per cent of total harvestable above ground biomass in winter wheat, and 59.9, 59.5, 56.1, 54.5, 54,2 and 54.8 per cent of total harvestable above ground biomass in maize for CK, N, NP, FYM, NP+S and NP+FYM, respectively. The stubble on an average constituted 10 per cent of the straw.

Root biomasses in winter wheat and maize were calculated using the root: shoot ratio. After harvest, four soil cores (8 cm diameter by 100 cm depth) per plot (two from rows and the other two from between rows) were collected from the 0 to 100 cm soil depths to measure root biomass. The root biomass represented 20.6, 23.6, 19.4, 20.6, 21.6, and 21.8 per cent of the harvestable above ground biomass in winter wheat, and 7.1, 8.0, 7.0, 8.9, 7.7, and 8.8 per cent of the harvestable above ground biomass in maize, respectively in the treatments listed above.

In 1997 and 2005, portions of air-dried straw, stubble and straw were passed through a 0.25 mm sieve for the determinations of the C concentrations. The root biomass, stubble and straw contained 40.4, 42.9 and 42.9 per cent C for winter wheat and 41.5, 44.6 and 44.6 per cent C for maize, respectively. While calculating total rhizodeposition derived from different crops in this study, we used the values mentioned by Bronson et al. [25]. Root exudates therefore represented 15% of aboveground biomass at maturity with a C concentration of 36% in CK and N treatments and 33% in NP, NP+S, FYM and NP+FYM treatments.

### Estimation of Soil Organic Carbon Stock

Total SOC stock of profile for each of the five depths (0–20, 20–40, 40–60, 60–80, 80–100 cm) was computed by multiplying the SOC concentration by the bulk density, depth, and factor by 10 (Equation 1).
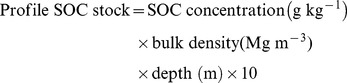
(1)


### Statistical Analysis

The effects of fertilizer treatments on SOC and labile SOC fractions (MBC, DOC, POC) within each depth were analyzed using one-way ANOVA. Differences were considered significant at P<0.05. Pearsons linear correlation were used to evaluate the relationships between SOC and POC, MBC and DOC. Linear-regression analyses were performed to determine trends using composite soil data from three replicate plots to assess trends of SOC (0–20 cm layer ) over the years. For statistical analysis of data, Microsoft Excel (Microsoft Corporation, USA) and SPSS window version 11.0 (SPSS Inc., Chicago, USA) packages were used. Unless otherwise stated, the level of significance referred to in the results is P<0.05.

## Results

### Soil Organic Carbon (SOC) Trends

**Figure 2:**
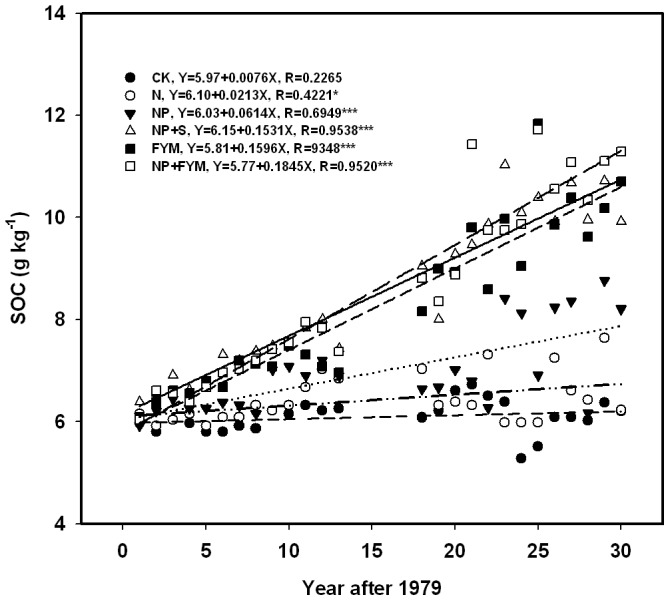
Trend changes of soil organic carbon (SOC) at 0–20 cm top soil layer in a long-term (1979–2008) fertilization experiment in Pingliang, Gansu, China.

The SOC concentrations in the 0–20 cm soil layer for CK, N, NP, FYM, NP+S and NP+FYM treatments at the beginning of the study in 1979 were 5.97, 6.15, 5.92, 6.38, 6.09, 6.03 g kg^−1^. Above data was the source of the growth rates. Although there were large fluctuations of SOC content with time, the SOC content in CK and N treatments generally constant with time and in NP, slightly increased (Fig. 2). The SOC concentration significantly increased with the lapse of year in the C input treatments (FYM, NP+S and NP+FYM). Across the 30 cropping and fertilization periods, annual SOC concentration rates (slopes of the linear regression vs. time) in Fig. 2 indicated that 0.15, 0.16 and 0.19 g kg^−1^ yr^−1^ were increased each year in NP+S, FYM and NP+FYM treatments, respectively.

### Bulk Density

**Figure 3:**
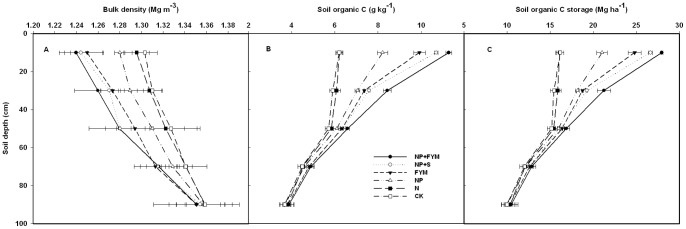
Effect of long-term fertilizer applications on depth distribution of bulk density (A), soil organic C (B) and soil organic C storage (C).

Long-term application of manure and fertilizer significantly affected soil bulk density (BD) to a depth of 40 cm (Fig. 3A). The addition of FYM or straw (FYM, NP+FYM and NP+S) treatments decreased soil bulk density significantly in comparison to that in control plots in all the layers. However, the decrease was more in upper soil layers (0–20 and 20–40 cm) than in the lower layers (40–60, 60–80 and 80–100 cm). Similar was the case with NP treatment, where BD was lower than that in CT treatment at 0–20 and 20–40 cm depths. There were no statistically significant differences in BD among treatments below 40 cm depth.

### Depth Distribution of Soil Organic Carbon

The distribution of SOC with depth was dependent on the use of various fertilizers (Fig. 3B). The highest SOC concentration was obtained for 0–20 cm depth and decreased with depth for all treatments. The SOC concentration in 0–20, 20–40 and 40–60 cm depths increased significantly by farmyard manure or straw application. At the 0–20 and 20–40 cm soil depths, SOC was highest in NP+FKM followed by NP+S and FYM treatments and the least in CK treatment. However, the SOC concentration below 60 cm depth was statistically similar among different treatments.

### Soil Organic Carbon Storage

The effects of fertilization on SOC storage showed a similar trend to SOC concentration (Fig. 3C). The topsoil (0–20 cm) had the maximum levels of cumulative SOC storage in the 1 m soil depth for the CK, N, NP, FYM, NP+S and NP+FYM treatments, accounting for 24%, 23%, 27%, 30%, 31% and 31%, respectively. At the 20–40 cm and 40–60 cm soil layers, the SOC stocks of the NP, FYM, NP+S and NP+FYM treatments were significantly higher by 17%, 21%, 25% and 37% and 5.3%, 8.1%, 7.3% and 11%, respectively, than that of the CK. The differences of SOC storage between different treatments were not significant in the 60–80 cm and 80–100 cm soil layers. SOC storages were significantly different between fertilization treatments in the 0–100 cm profile. Compared with the CK treatment, SOC storages of the NP+FYM, NP+S, FYM and NP treatments within the 0–100 cm soil depth were increased by nearly 30, 24, 20 and 12%, respectively.

### Particulate Organic Carbon

**Figure 4:**
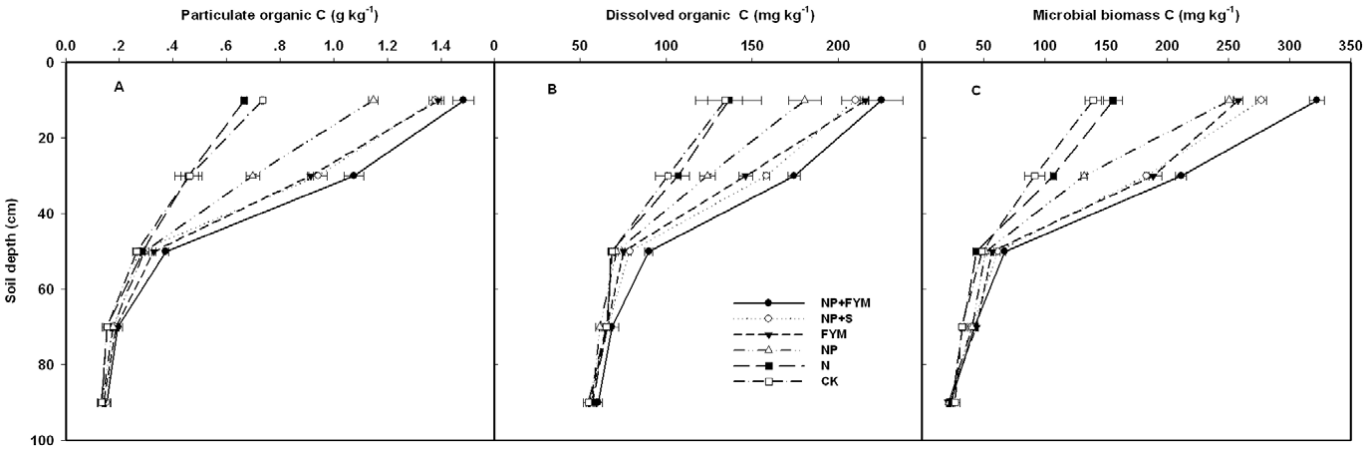
Effect of long-term fertilizer applications on depth distribution of particulate organic C (A), dissolved organic C (B) and microbial biomass C (C).

Particulate organic C was found stratified along the soil depth. A higher POC was found in surface soil decreasing with depth (Fig. 4A). At the 0–20 cm, POC content under NP+FYM, NP+S and FYM were 103, 89 and 90% greater than under CK, respectively. In 20–40 cm and 40–60 cm soil layers, NP+FYM had maximum POC which was significantly higher than NP+S and FYM treatments. Even though POC below 60 cm depth was statistically similar among fertilization treatments, the general trend was for increased POC with farmyard manure or straw application down to 100 cm soil depth.

### Dissolved Organic Carbon

Irrespective of soil depths, NP+FYM invariably showed higher content of DOC over all other treatments. The CK and N treatments showed lower content of DOC. The DOC concentrations in 0–20 cm, 20–40 cm and 40–60 cm depths were observed highest for NP+FYM followed by NP+S and FYM, and both of them were significant higher than NP (Fig. 4B). However, in the deeper layers (60–80 cm and 80–100 cm), the difference in DOC among the treatments was not significant.

### Microbial Biomass Carbon

The MBC differences among treatments not only presented in the surface soil layers, but also presented at deeper depths in the profile. In our study, MBC showed a significant effect at different fertilizer treatments (Fig. 4C). The SOC concentration in 0–80 cm depth increased significantly by farmyard manure or straw application. The mean MBC content in 0–80 cm profile was 82% higher in NP+FYM treatment than in CK treatment.

### Comparison of Labile Organic Carbon Pools

**Figure 5:**
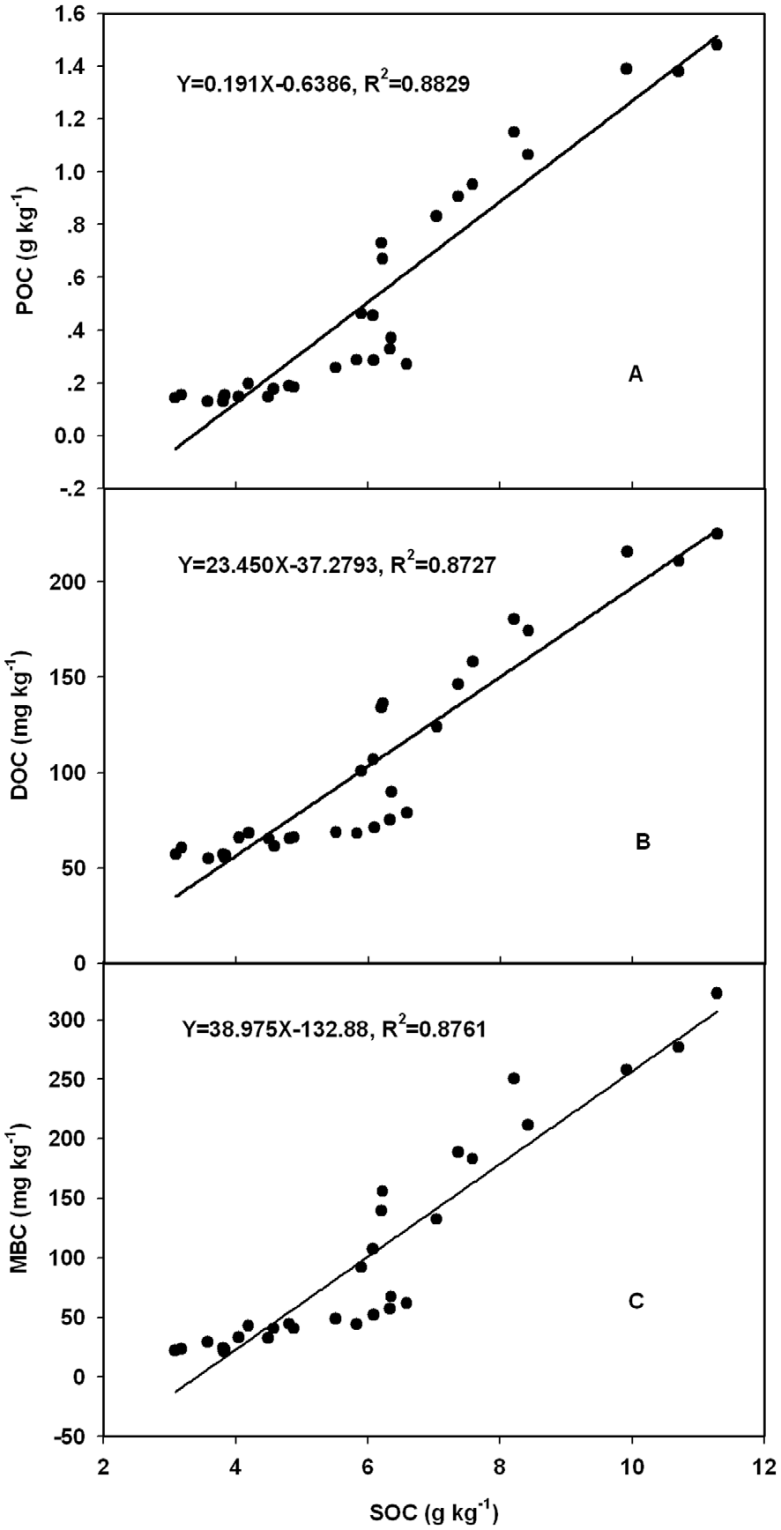
Relationship between soil organic carbon and different labile SOC pools: (A) dissolve organic C (DOC), (B) particulate organic C (POC), and (C) microbial biomass C (MBC).

POC, MBC and DOC concentrations increased linearly with increasing soil SOC content (Fig. 5), suggesting that total organic matter content was a major determinant of the amount of POC, MBC and DOC present. Of the reported C pools, POC was most highly correlated with SOC, followed by MBC then DOC.

## Discussion

### Soil Total C

From 1979 to 2008, SOC concentrations (0–20 cm layer ) increased significantly for all treatments except the CK and N treatments, and the greatest increases occurred for the three plots of FYM, NP+FYM and NP+FYM treatments that received organic materials (Fig. 2). Apparently, application of only N fertilizer did not increase SOC content over long-term cropping. This observation was consistent with that of Goyal et al. [26], who reported that no significant increase in SOC by the addition of only N fertilizer. In the present study, SOC in the CK and N soil were at par, presumably because of lower crop productivity that results in significantly lower accumulation of root biomass. The NP treatment increased SOC concentrations but at a much lower rate of 0.061 g kg^−1^ yr^−1^. This finding indicated that long-term chemical NP fertilizer alone can increase soil C sequestration, which has been confirmed by other long-term fertilizer experiments in China [27]. A before linear relationship was found between SOC and organic material (i.e., manure and straw) input after 30 y of continuous cropping in northwest China. Most of the soil organic matter (SOM) models assume a linear increase in SOC levels with increasing C input [28].

The SOC concentration differences among treatments not only presented in the surface soil layers, but also presented at deeper depths in the profile. SOC concentration was highest in NP+FYM plots and the least in unfertilized control (CK) at all the sampling depths. Though the average SOC concentration decreased with soil depth, the NP+FYM, NP+S and FYM treatments resulted in a significant increase in organic C even in 40–60 cm soil layer. Manjaiah and Singh [29] and also found that inorganic fertilizers plus organic material increased the SOC content of the soil. The reasons for the higher SOC in manure soils at deeper depths include the following. First, the crop rooting depth between organic manure and inorganic fertilizer soils differ. The organic manure soils can be favorable for the growth of roots into deeper layers due to the relatively loose soil and high soil water content. Second, SOC in organic manure soils can also move to lower depths through earthworm burrows and leaching [30]. Applying straw with N and P fertilizer (NP+S) had the highest total C input (Table 1), yet decreased SOC concentration over the FYM with NP fertilizer treatment. As NP+FYM treatment significantly increased SOC concentration, this suggests that animal manure is more effective in building soil C than straw, possibly due to the presence of more humified and recalcitrant C forms in animal manure as compared to the straw. For the inorganic fertilizer treatment, the optimum application of inorganic fertilizer NP treatment showed a higher SOC concentration over the application of inorganic fertilizer N treatment at all the sampling depths. The optimum fertilization results in better plant growth including the root biomass (Table 1), which could have added to the SOC particularly as indicated in the lower layers [31].

Similar to the concentration, SOC stock of the profile was also significantly (*P*<0.05) higher in the organic manure treatments (FYM, NP+FYM and NP+S) compared with the only inorganic fertilizer (N, NP) and control treatments. Gami et al. [32] also reported a significant increase in SOC stocks to 60 cm depth under three 23–25-year-old long-term fertility experiments in the Nepal, with application of manure and inorganic fertilizer. Within 1 m soil depth, the cumulative distribution of SOC in the CK, N, NP, FYM, NP+S and NP+FYM treatments were by 50%, 46%, 51%, 53%, 54% and 55% in the 0–40 cm layer, and 68%, 68%, 71%, 72%, 73% and 74% in the 0–60 cm layer, respectively. The SOC storage in the 60–100 cm layer was statistically similar among different treatments. On average the estimate of soil C accumulation to 60 cm depth were 267% and 41% greater than that for soil C accumulated to 20 cm depth and to 40 cm depth, respectively. These findings suggest that the estimate of soil C accumulation to 60 cm depth was more effective than that for soil C accumulated to 40 cm. In this study, C input was increased under the N treatment compared to the CK. However, neither SOC concentration nor C storage was significantly changed under the N treatment. The reason for this is that the N treatment may stimulate soil microbial activity, therefore increasing the C output. The increase in C mineralization might offset the increase in C input. Similar results were also found by Halvorson et al. [33], Su et al. [34], and Lou et al. [35].

### Soil C Fractions

The POC fraction has been defined as a labile SOC pool mainly consisting of plant residues partially decomposed and not associated with soil minerals [23], [36]. In the present study, the soil amended by FYM or straw contained significantly higher POC in the 0–60 cm than that in the inorganic fertilizer treatments. Rudrappa et al. [31] reported that the additional organic carbon input could enhance the POC accumulation. Purakayastha et al. [5] concluded that FYM can increase the root biomass and microbial biomass debris which is the main source of POC. It is suggested that the greater biochemical recalcitrance of root litter [37] might have also increased the POC contents in soil depending upon the root biomass produced. The continuous replacement of organic manure on the soil creates a favourable environment for the cycling of C and formation of macroaggregates. Furthermore, POC acts as a cementing agent to stabilise macroaggregates and protect intra-aggregate C in the form of POC [36], [38]. Below 60 cm soil layer, the POC declined with increase in soil depth. Chan [39] also found that straw application increased POC in surface soil but not at lower depths.

DOC is believed to be derived from plant roots, litter and soil humus and is a labile substrate for microbial activity [40], [41]. The concentration of DOC varied widely among all the treatments and a significant increase was observed in surface soils under different fertilizer treatments compared with CK. In the long-term, the quantity of organic residues are the main factors influencing the amount and composition of DOC. Likewise, in our study, the upper 60 cm soil layer had more DOC concentration than that of lower layer. Below 60 cm, the DOC concentration sharply decreased with soil depth. DOC in subsurface soils may be a result of decomposition of crop residues or translocation from surface soil [42]. Several field studies have sown that concentration and fluxes of DOM in soil solution decrease significantly with soil depth [41].

In our study, MBC was highest in the farmyard manure plus inorganic fertilizer treatment in top soil, an increased MBC content after farmyard application was also reported by Chakraborty et al. [43] and Marschner et al. [44]. This indicated the activation of microorganisms through carbon source inputs consisting of organic residues. Increases in soil organic matter are usually associated with similar increases in microbial biomass because the SOM provides principal substrates for the microorganisms [45]. Among the investigated fertilizer treatments, straw plus inorganic fertilizer had impact on the microbial biomass. This effect is mainly due to the input of straw manure as an organic carbon source. Lynch and Panting [46] reported that eight months after application of straw manure to a loamy arable soil the microbial biomass was almost twice as high as compared to a control. Also, Ocio et al. [47] have demonstrated rapid and significant increases in microbial biomass following straw inputs in field conditions. The MBC was not only correlated with SOC concentration near the surface but also at deeper depths. Though the average MBC decreased with soil depth, the NP+FYM, NP+S and FYM treatments resulted in significant increase in microbial biomass C even in 60–80 cm soil layer. The main source of MBC in deep soil was mainly the left over root biomass and increased microbial biomass debris. It is suggested that the greater biochemical recalcitrance of root litter [37] might have also increased the MBC contents in soil depending upon the root biomass produced. However, the MBC content was lower in the 80–100 cm profile. The reason is that roots may be difficult extend to lower depths. The imbalanced use of fertilizers (CK and N) decreased MBC due to limitation imposed by major nutrients like P and K, which are essential for higher crop production as well as for microbial cell synthesis.

SOC was highly correlated with labile carbon (POC, MBC and DOC). POC, MBC and DOC were significantly and positively correlated with SOC. The correlation coefficient was highest between POC and SOC (R^2^ = 0.883), followed between MBC and SOC (R^2^ = 0.876) and DOC and SOC (R^2^ = 0.873). Such high correlations have also been reported by Rudrappa et al. [31] and Liang et al. [12], and it is not surprising that the three measures of labile organic matter were closely correlated since they are closely interrelated properties. This result confirms the value of these fractions as sensitive indicators for detecting changes in SOM in the short term, before they are readily measurable in total C. Likewise, these correlations also indicated that SOC was a major determinant of the labile C fractions present.

### Conclusions

Fertilizer application has played an important role in improving the total SOC and labile C pools content in the soil after 30 years. Because there was low SOC content in the Northwest of China, the long-term application of organic manure and inorganic fertilizer increased the content of SOC. SOC concentrations and storage were highest in surface soil and depth interval down to 60 cm under NP+FYM and NP+S, below which concentrations did not change with depth. At the same time, on average the estimate of soil C storage to 60 cm depth was higher than that for soil C accumulated to 20 cm depth and to 40 cm depth, respectively. These findings suggest that the estimate of soil C accumulation to 60 cm depth was more effective than that for soil C accumulated to 20 cm depth and to 40 cm depth. NP+FYM was the most efficient management system for sequestering SOC. A large amount of C was also sequestered in soil under NP+S treatment. Soil microbial biomass C, POC and DOC were all significantly greater under organic manure (farmyard manure or straw) plus inorganic fertilizers, especially in the surface. The labile fraction organic C contents decreased significantly with increasing soil depth. These labile pools were highly correlated with each other and SOC, indicating that they were sensitive to changes in SOC.

In Northwest China, the effects of manure and fertilizer application practices on soil C sequestration were studied so that dryland farming soil could contribute to both sustainable food production and mitigation of greenhouse gas emissions through soil C sequestration. Our results have very significant implications for soil C sequestration potential in semiarid agro-ecosystems of northwest China. SOC concentration in surface soil (0–20 cm) and SOC storage of the profile (0–100 cm) were not significantly or slightly increased by the 30 yr of fertilizer treatments (N and NP), but they were sharply increased by the manure and straw amendment (FYM, NP+S and NP+FYM). Thus, returning crop residue to the soil or adding farmyard manure on the soil surface is crucial to improving the SOC level. The large scale implementation of the straw or manure plus inorganic fertilizer amendments will help to enhance the capacity of carbon sequestration and promote food security in the region. Therefore, local government should encourage farmers to manage the nutrients and soil fertility based on integrated nutrient management by combining organic matter with inorganic fertilizer to improve soil carbon pools and increase crop productivity for long-term.
